# A Microarray-Based Gene Expression Analysis to Identify Diagnostic Biomarkers for Unknown Primary Cancer

**DOI:** 10.1371/journal.pone.0063249

**Published:** 2013-05-09

**Authors:** Issei Kurahashi, Yoshihiko Fujita, Tokuzo Arao, Takayasu Kurata, Yasuhiro Koh, Kazuko Sakai, Koji Matsumoto, Maki Tanioka, Koji Takeda, Yuichi Takiguchi, Nobuyuki Yamamoto, Asuka Tsuya, Nobuaki Matsubara, Hirofumi Mukai, Hironobu Minami, Naoko Chayahara, Yasuhiro Yamanaka, Keisuke Miwa, Shin Takahashi, Shunji Takahashi, Kazuhiko Nakagawa, Kazuto Nishio

**Affiliations:** 1 Department of Planning, Information, and Management, University of Tokyo, Tokyo, Japan; 2 Department of Genome Biology, Kinki University School of Medicine, Osaka-Sayama, Japan; 3 Department of Medical Oncology, Kinki University School of Medicine, Osaka-Sayama, Japan; 4 Division of Drug Discovery and Development, Shizuoka Cancer Center Research Institute, Shizuoka, Japan; 5 Medical Oncology Division, Hyogo Cancer Center, Akashi, Japan; 6 Department of Clinical Oncology, Osaka City General Hospital, Osaka, Japan; 7 Department of Medical Oncology, Graduate School of Medicine, Chiba University, Chiba, Japan; 8 Division of Thoracic Oncology, Shizuoka Cancer Center, Shizuoka, Japan; 9 Division of Oncology and Hematology, National Cancer Center Hospital East, Chiba, Japan; 10 Division of Medical Oncology/Hematology, Kobe University Graduate School of Medicine, Kobe, Japan; 11 Department of Medical Oncology, Tochigi Cancer Center, Utsunomiya, Japan; 12 Department of Medical Oncology, International Medical Center-Comprehensive Cancer Center, Saitama Medical University, Saitama, Japan; 13 Department of Clinical Oncology, Institute of Development, Aging and Cancer, Tohoku University, Sendai, Japan; 14 Division of Medical Oncology, Cancer Institute Hospital, Tokyo, Japan; Queensland University of Technology, Australia

## Abstract

**Background:**

The biological basis for cancer of unknown primary (CUP) at the molecular level remains largely unknown, with no evidence of whether a common biological entity exists. Here, we assessed the possibility of identifying a common diagnostic biomarker for CUP using a microarray gene expression analysis.

**Methods:**

Tumor mRNA samples from 60 patients with CUP were analyzed using the Affymetrix U133A Plus 2.0 GeneChip and were normalized by asinh (hyperbolic arc sine) transformation to construct a mean gene-expression profile specific to CUP. A gene-expression profile specific to non-CUP group was constructed using publicly available raw microarray datasets. The t-tests were performed to compare the CUP with non-CUP groups and the top 59 CUP specific genes with the highest fold change were selected (*p*-value<0.001).

**Results:**

Among the 44 genes that were up-regulated in the CUP group, 6 genes for ribosomal proteins were identified. Two of these genes (*RPS7* and *RPL11*) are known to be involved in the Mdm2–p53 pathway. We also identified several genes related to metastasis and apoptosis, suggesting a biological attribute of CUP.

**Conclusions:**

The protein products of the up-regulated and down-regulated genes identified in this study may be clinically useful as unique biomarkers for CUP.

## Introduction

Patients with cancer of unknown primary (CUP) present with metastatic disease for which the primary site cannot be found, despite extensive standard investigation. The prognosis of patients with CUP is usually poor for those receiving empiric treatments. The median survival period is 3–9 months even when newer combination treatment regimens are administered [1]–[5]. The survival of patients with CUP can be improved if the primary site can be identified and a site-specific therapy can be applied [6], [7].

Clinically, CUPs exhibit common characteristics, such as rapid progression, early dissemination and a silent primary tumor, with signs and symptoms related to the metastatic site(s) [8]. The primary tumor may either have a slow growth pattern or may become involuted and undetectable. Existence of such common properties prompts us to hypothesize that there may be potential biological markers that elucidate CUP as a whole. Gene expression analysis is one of the means by which to identify genes characteristic to CUP.

Several studies using gene expression microarrays have demonstrated that the expression levels of thousands of genes can be used as a “molecular fingerprint” to classify a multitude of tumor types [9]–[15]. We are presently involved in a multicenter clinical study to predict the primary site of CUP based on the analysis of gene expression patterns. The analysis interprets the expression of ∼22,000 genes in each specimen by applying normalization and classification algorithms to gene expression data from a microarray. The similarity of each tumor specimen's gene expression pattern is then compared to the patterns for tumors from 24 known primary sites covered by the test. This study enabled the identification of genes that exhibited a unique expression pattern in CUP. Here, we present several genes encoding metastasis-and apoptosis-related proteins thus identified that may biologically characterize CUP.

## Materials and Methods

### Ethic Statement

All the patients provided written informed consent. Study approval was obtained from independent ethics committees of Kinki University, Shizuoka Cancer Center, Hyogo Cancer Center, Osaka City General Hospital, Chiba University, National Cancer Center Hospital East, Kobe University, Tochigi Cancer Center, Saitama Medical University, Tohoku University, and Cancer Institute Hospital. The study was undertaken in accordance with the Declaration of Helsinki.

### Study Design

This study originated from currently ongoing multicenter, randomized, phase 2 prospective trial for the treatment of untreated CUP based on prediction of the primary site using data from a DNA chip. The patients had been diagnosed as having CUP between November 2008 and November 2010 at one of 13 centers of the West Japan Oncology Group (WJOG), a Japanese non-profit organization for conducting oncological clinical trials. The laboratory analyses were performed at 2 centers in Japan (Kinki University, Osaka-Sayama and Mitsubishi Chemical Medience Corporation, Tokyo).

### Patients

All eligible patients had undergone a standard investigation for CUP. They were categorized into unfavorable subsets of CUP. Diagnoses of histologically or cytologically confirmed adenocarcinoma, poorly differentiated carcinoma, or squamous cell carcinoma were permitted. In each of the patients, a primary site had not been identified after a complete medical history, physical examination, chemistry profile, computed tomography (CT) scan of the chest, abdomen, and pelvis, mammography in women, measurements of the prostate-specific antigen (PSA) level in men, and a directed workup of any symptomatic areas. Patients in the following categories were excluded: women with adenocarcinoma involving only the axillary lymph nodes or the peritoneal cavity, patients with squamous cell carcinoma involving only cervical lymph nodes or inguinal lymph nodes, patients with poorly differentiated carcinoma consistent with a germ cell tumor (isolated midline structures, multiple pulmonary nodules, or elevated levels of β-human chorionic gonadotropin or α-human chorionic gonadotropin-fetoprotein), men with an elevated plasma PSA level or PSA-positive staining in a tumor, patients with a single, small, potentially resectable tumor, and patients with neuroendocrine carcinomas.

### Sample Collection

Fresh frozen samples obtained from 60 patients with CUP were used for the analysis. All the samples were tested without knowledge of either the clinical characteristics or the subsequent response to treatment, except for the sex of the patient and the site of biopsy (mostly lymph nodes or ascites fluid).

### Assay Procedure

RNA was extracted from the samples using an Isogene kit (Nippon Gene, Toyama, Japan). Spectrophotometry was used to assess whether an adequate total RNA concentration and purity was present. In general, the protocol for processing the RNA, amplifying and labeling fragments, hybridizing material on the microarray, and scanning was similar to the standard Affymetrix protocol for GeneChip® expression analysis. Affymetrix GeneChip® Human Genome U133 Plus 2.0 was used on an Affymetrix 3000 or 3000Dx GeneChip instrument (fluidics station and scanner) running Gene-Chip operating software to generate gene expression data (.CEL files).

### Database Submission of Microarray Data

The microarray data were deposited in the Gene Expression Omnibus (GEO) database: http://www.ncbi.nlm.nih.gov/geo/. The GEO accession number for the platform is GSE42392, samples GSM1038716-GSM 1038775.

### Data Analysis

All the microarray data were normalized using asinh (hyperbolic arc sine) transformation, which is a modified version of Huber's normalization with variance stabilization [16], [17], and also a part of generalized log transformation (glog) [18]. Interinstitutional and array-to-array biases were corrected by subtracting their specific effects that were estimated by the mixed model [19]. The equation for asinh transformation is Igk/I.k, where I represents the expression value, g represents the gene, k represents the array, and the dot indicates the mean. The resulting asinh-transformed values, representing the relative expression of each gene, were used in further analyses.

The raw microarray datasets for 2,364 cancers of several primary types and 10 normal lymph nodes were obtained from the Gene Expression Omnibus (GEO) (Table 1). These datasets were normalized and used to construct gene-expression profiles specific to each type of cancer (n = 24) as well as an overall profile for cancer with known primary (CKP). The normal lymph node dataset was used as a reference. The data quality of CUP samples was monitored to ensure that data analysis of CUP samples was comparable to that of samples of CKP collected from GEO. Only the samples whose GAPDH, a housekeeping control gene, at 5′-terminal region (AFFX-HUMGAPDH/M33197_5_at) showed a minimum expression>500, and with the ratio of expression intensity (GAPDH at the 3′-region/5′-region)<3 were chosen.

**Table 1:** Number of cases for each cancer type and GEO series used for gene expression profiles.

Cancer type	n	GEO Series						
Bladder	80	GSE2109, GSE3167, GSE7476						
Brain	106	GSE2109, GSE3185, GSE4271						
Breast (Basal)	25	GSE1456						
Breast (ERBB2)	15	GSE1456						
Breast (Inflammatory)	49	GSE1456						
Breast (Luminal A)	39	GSE1456						
Breast (Luminal B)	23	GSE1456						
breast (No subtype)	20	GSE1456						
Breast (Normal-like)	37	GSE1456						
Cervical	89	GSE2109, GSE5787, GSE6791						
Colon	365	GSE2109, GSE2509, GSE2742, GSE5486, MEXP101, MEXP170						
Corpus_uteri	205	GSE2109						
Gallbladder	2	GSE2109						
Germ cell	101	GSE3218						
Head (oral squamous cell)	42	GSE6791						
Kidney	270	GSE2109, GSE6357, GSE781						
Liver	13	GSE2109						
Lung adenocarcinoma	61	GSE4127, MEXP231						
Lymphoma	18	GSE2109, GSE4176						
Ovarian	420	GSE2109, GSE3149						
Panreas	56	GSE2109						
Prostate	229	GSE2109, GSE3325, GSE7930, GSE8218						
Stomach	42	GSE2109						
Thyroid	57	GSE2109, MEXP97						
Normal lymph node	10	GSE2665						
CUP (This work)	60	GSE42392						
**Total**	**2434**							

**Figure 1:**
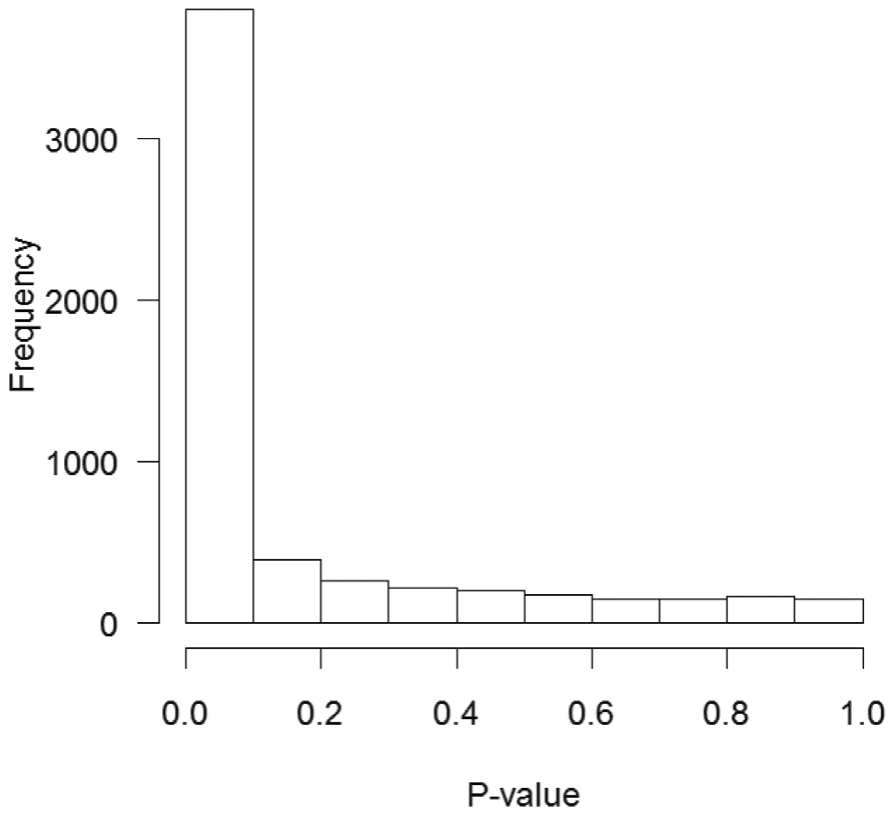
Frequency histogram of *p*-values.

The gene-expression profile specific to CUP was constructed using 30 CUP samples as training data and another 30 samples as test data (odd and even numbered cases, respectively). Of the 22,215 genes that were measured using both CUP samples (this work) and CKP samples (publicly accessed), a total of 5,645 genes with a present call for every sample were selected for further analysis. To identify CUP specific genes, the gene-expression profiles specific to CUP (training datasets) and normal lymph node were compared using t-tests. A histogram of the *p*-values is shown in Figure 1. The *p*-values for most of the genes were less than 0.001; when we selected the top 100 genes according to their *p*-values, the false discovery rate (FDR) was 4.56×10^−12^ [20]. To validate whether the genes identified using the CUP training datasets were significantly specific to CUP, the linear discriminant analysis (LDA) using these genes was performed for the CUP test datasets and the accuracy was estimated as described [21]. Heatmaps and a cluster dendrogram were then constructed using the Ward method [22].

## Results

### Gene Expression Profile of CUP and Known Primary Cancers

A total of 237 genes were found to be either up-regulated or down-regulated by more than 2-fold between the normal lymph node and 30 CUP samples (training datasets). Of these, 59 genes with more than a 2.5-fold change (44 up-regulated and 15 down-regulated genes) are listed in Table 2. We designated the gene sets consisting of these CUP associated genes with >2 fold and >2.5 fold up-regulation or down-regulation as M_CUP_(2.0) and M_CUP_(2.5), respectively. Using these probe sets in M_CUP_(2.5), linear discriminant analysis (LDA) was performed for the CUP training datasets together with 2,364 cancers of various known types and 10 normal lymph nodes. As expected, all 2,404 samples were correctly discriminated. When the remaining 30 CUP samples (test datasets) were assessed using LDA that was modeled with the training datasets, 26 out of the 30 CUP samples were assigned correctly to “CUP”, while only the 4 samples were predicted as "the other cancer". Thus, the accuracy of CUP was validated to be 86.7%, indicating that the 59 genes selected were of statistically significance as having biological attributes of CUP.

**Table 2:** Genes identified as being up-regulatred or down-regulated in CUP.

Symbol	Gene description (Gene up-regulated in CUP)	Probe_set_ID	Log-fold change*	Fold change
RPL18A	Ribosomal protein L18A	200869_at	1.974	7.2
S100A4	S100 calcium binding protein A4	203186_s_at	1.587	4.9
PRG1	Proteoglycan 1, secretory granule	201858_s_at	1.539	4.7
SUB1	SUB1 homolog (S. cerevisiae)	214512_s_at	1.535	4.6
S100A6	S100 calcium binding protein A6	217728_at	1.523	4.6
RPS7	Ribosomal protein S7	200082_s_at	1.369	3.9
RPL11	Ribosomal protein L11	200010_at	1.245	3.5
PFN1	Profilin 1	200634_at	1.229	3.4
LOC23117	KIAA0220-like proteinKIAA0220	211996_s_at	1.212	3.4
TYROBP	TYRO protein tyrosine kinase binding protein	204122_at	1.196	3.3
TIMP1	TIMP metallopeptidase inhibitor 1	201666_at	1.178	3.2
SERF2	Small EDRK-rich factor 2	217756_x_at	1.173	3.2
YWHAZ	14-3-3 protein, zeta polypeptide	200641_s_at	1.169	3.2
LSM7	LSM7 homolog, U6 small nuclear RNA associated (S. cerevisiae)	204559_s_at	1.151	3.2
GSTP1	Glutathione S-transferase pi	200824_at	1.141	3.1
YWHAHLAPTM5	14-3-3 protein, eta polypeptideLysosomal associated multispanning membrane protein 5	201020_at201721_s_at	1.1021.095	3.03.0
SNRPD2	Small nuclear ribonucleoprotein D2 polypeptide 16.5 kDa	200826_at	1.087	3.0
LOC392501	similar to 60 S ribosomal protein L26	222229_x_at	1.076	2.9
OAZ1	Ornithine decarboxylase antizyme 1	215952_s_at	1.073	2.9
POLR2J	Polymerase (RNA) II (DNA directed) polypeptide J, 13.3kDa	212782_x_at	1.062	2.9
EIF5A	Eukaryotic translation initiation factor 5A	201123_s_at	1.028	2.8
ATP5H	ATP synthase, H+ transporting, mitochondrial F0 complex, subunit d	210149_s_at	1.023	2.8
APOC1	Apolipoprotein C-I	213553_x_at	1.018	2.8
LGALS1	Lectin, galactoside-binding, soluble, 1 (galectin 1)	201105_at	1.013	2.8
S100A11	S100 calcium binding protein A11	200660_at	1.010	2.7
SH3BGRL3	SH3 domain binding glutamic acid-rich protein like 3	221269_s_at	0.996	2.7
C1QB	complement component 1, q subcomponent, B chain	202953_s_at	0.984	2.7
RPS10	Ribosomal protein S10	216505_x_at	0.984	2.7
HSPA8	Heat shock 70 kDa protein 8	210338_s_at	0.972	2.6
NUTF2	Nuclear transport factor 2	202397_at	0.972	2.6
PRKDC	Protein kinase, DNA-activated, catalytic polypeptide	208694_at	0.967	2.6
NOLA3	Nucleolar protein family A, member 3 (H/ACA small nucleolar RNPs)	217962_at	0.957	2.6
TCEB2	Transcription elongation factor B (SIII), polypeptide 2 (18 kDa, elongin B)	200085_s_at	0.953	2.6
LOC442171	similar to ribosomal protein L10	217379_at	0.952	2.6
NEDD8	Neural precursor cell expressed, developmentally down-regulated 8	201840_at	0.944	2.6
LOC646417	similar to 60 S ribosomal protein L29 (P23)	216570_x_at	0.939	2.6
RPL36	Ribosomal protein L36	219762_s_at	0.937	2.6
VIM	Vimentin	201426_s_at	0.924	2.5
STK17A	Serine/threonine kinase 17a (apoptosis-inducing)	202693_s_at	0.922	2.5
NDUFS8	NADH dehydrogenase (ubiquinone) Fe-S protein 8, 23 kDa	203189_s_at	0.911	2.5
SELT	Selenoprotein T	217811_at	0.908	2.5
CST3	Cystatin C (amyloid angiopathy and cerebral hemorrhage)	201360_at	0.906	2.5
RPLP2	Ribosomal protein, large, P2	200909_s_at	0.901	2.5
Symbol	Gene description (Gene down-regulated in CUP)	Probe_set_ID	Log-fold change*	Fold change
ATP1B1 NGFRAP1	ATPase, Na+/K+ transporting, beta 1 polypeptideNerve growth factor receptor (TNFRSF16) associated protein 1	201242_s_at 217963_s_at	−0.891 −0.968	0.4 0.4
FOXJ3	Forkhead box J3	206015_s_at	−0.978	0.4
GABARAPL1	GABA(A) receptor-associated protein like 1	211458_s_at	−0.984	0.4
CD24	CD24 molecule	216379_x_at	−0.995	0.4
IVNS1ABP	Influenza virus NS1A binding protein	206245_s_at	−1.000	0.4
SCAMP1	Secretory carrier membrane protein 1	212417_at	−1.037	0.4
SEC22B	SEC22 vesicle trafficking protein homolog B (S. cerevisiae)	214257_s_at	−1.047	0.4
ITM2B	Integral membrane protein 2B	217731_s_at	−1.071	0.3
PDIA3	Protein disulfide isomerase family A, member 3	208612_at	−1.071	0.3
PIN4	Protein (peptidylprolyl cis/trans isomerase) NIMA-interacting, 4 (parvulin)	214224_s_at	−1.087	0.3
KRAS	v-Ki-ras2 Kirsten rat sarcoma viral oncogene homolog	214352_s_at	−1.175	0.3
DICER1	Dicer1, Dcr-1 homolog (Drosophila)	213229_at	−1.264	0.3
SWAP70	SWAP-70 protein	209306_s_at	−1.342	0.3
VAPA	VAMP (vesicle-associated membrane protein)-associated protein A, 33 kDa	208780_x_at	−2.720	0.1

Each of the gene symbols, description, probe set in HG-U133 plus 2.0, log fold change and fold change are given in the table.

* Natural logarithm of fold change (CUP/normal lymph node).

**Figure 2:**
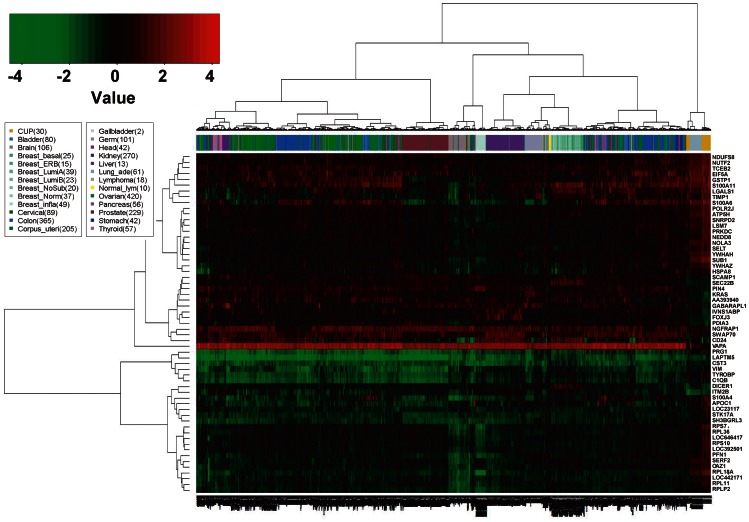
Heatmap representing the expressions of 59 genes with significant different expression in CUP compared with other cancer types or normal lymph nodes. Genes are indicated on the right. The colored bar above the heatmap represents the different cancer types, and the legend key is on the left. On the heatmap, red represents up-regulated genes and green represents down-regulated genes, relative to the expression levels in normal lymph nodes, with the scale shown in the upper left corner. The gene expression profiling datasets for normal lymph nodes and 24 known cancer types other than CUP were obtained from publicly available sources, as described in the Materials and Methods.

Figure 2 shows the supervised clustering of all 60 CUP samples performed together with 2,364 cancers of various known types and 10 normal lymph nodes using the 59 genes. The CUP samples were split into 2 groups with lung adenocarcinoma (LAC) clustered in between (right most part of the heat map). The larger group consisted of 42 samples, while the smaller consisted of 15 samples. Only 3 CUP samples were not included in any of these groups and instead were included in the clusters for normal lymphoma, brain tumors, and ovarian cancer, respectively. These were among the 4 samples that were predicted as “the other cancer” in the LDA. The *VAPA* gene, which was overexpressed in most of the cancer samples but not in CUP or LAC, revealed a striking contrast between CUP/LAC and other samples, which may have influenced the clustering analysis. When we re-analyzed the data after excluding the *VAPA* gene, the grouping for CUP was unchanged, but the smaller group with 15 samples was no longer clustered with LAC (Figure S1). The mean gene expression profiles (GEPs) for CUP, normal lymphoma, and 24 known cancer types were compared to create a dendrogram representing the quantified relations among CUP and the known cancer types, which again showed the clustering of CUP together with LAC (Figure S2).

### Selection of CUP Associated Genes

Although the functions were diverse or unknown for the 44 up-regulated genes in the M_CUP_(2.5) datasets (Table 2), we found that 14 genes (*S100A4*, *PRG1*, *S100A6*, *GSTP1*, *EIF5A*, *LGALS1*, *S100A11*, *PRKDC*, *VIM*, *CST3*, *TIMP1*, *YWHAZ*, *NEDD8*, *STK17A*) could be characterized after a search using the keywords “metastasis” and “apoptosis”. Some of these genes were associated with the epithelial-to-mesenchymal transition (EMT), a function that has been increasingly recognized as a key step in cancer metastasis [23].

In the M_CUP_(2.5) dataset, 15 genes were down-regulated. Of these genes, we focused on *CD24*, *KRAS* and *DICER1*. The known functions of the above-mentioned up-regulated and down-regulated genes will be discussed in detail below.

### Relative Expression of Up-Regulated Ribosomal Proteins

In the M_CUP_(2.5) dataset, we also identified 6 ribosomal proteins (*RPL18A*, *RPS7*, *RPL11*, *RPS10*, *RPL36*, *and RPLP2*). We found 11 more genes for ribosomal proteins (*RPL24*, *RPL35*, *RPL35A*, *RPS20*, *RPL13A*, *RPL28*, *RPS26*, *RPS14*, *RPL27A*, *RPL19*, *and RPL29*) in the M_CUP_(2.0) dataset. Ribosomal proteins are assembled into small and large ribosomal subunits. The small 40 S and large 60 S ribosomal subunits contain approximately 32 and 47 ribosomal proteins (known as RPS and RPL proteins), respectively [24]. The increased expression of ribosomal proteins has been associated with increased proliferation and growth; in some cases, however, increased expression has also been shown to suppress tumorigenesis [25], [26].

**Figure 3:**
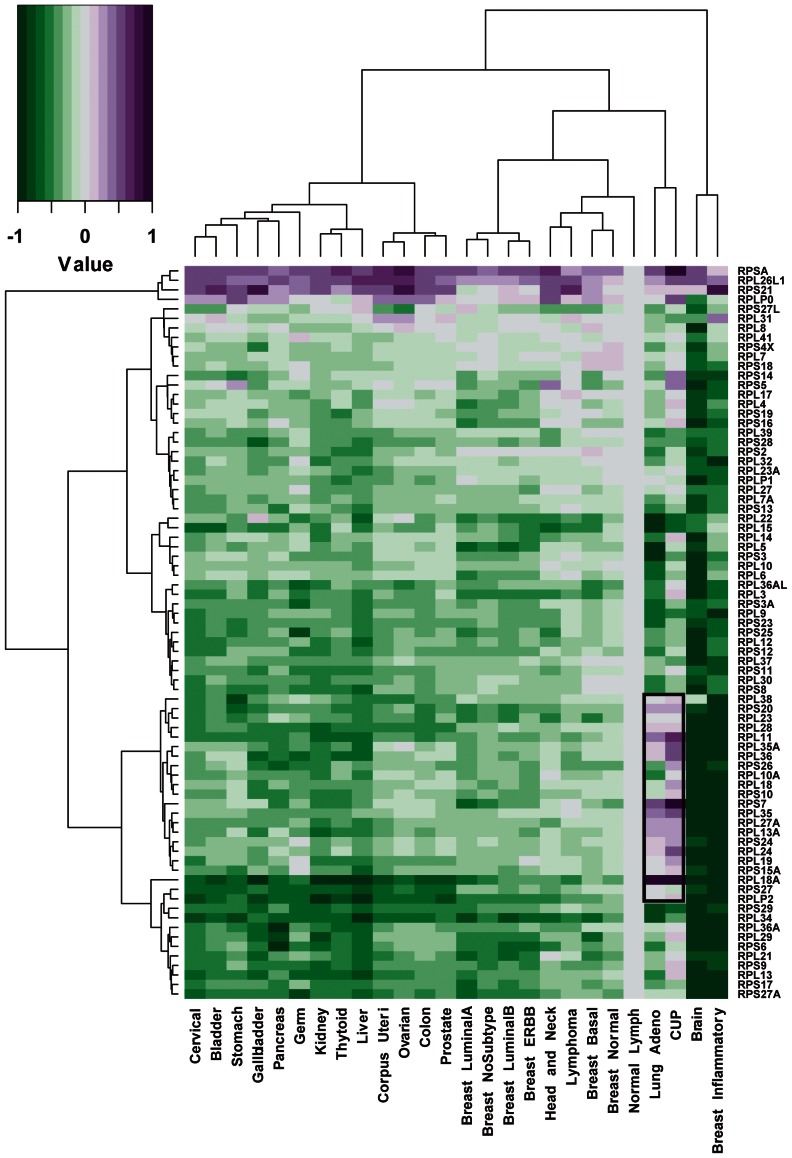
Heatmap representing the expression of 77 ribosomal protein genes in CUP, normal lymph nodes, and other cancer types. Ribosomal protein genes are indicated on the right. On the heatmap, purple represents up-regulated genes and green represents down-regulated genes, relative to the expression levels in normal lymph nodes, with the scale shown in the upper left. The genes that were exclusively overexpressed in CUP and lung adenocarcinoma are highlighted.

To examine whether ribosomal protein genes can be used as biomarkers to discriminate CUP from other cancer types, the mean GEPs for a total of 77 ribosomal protein genes were compared using clustering for CUP, normal lymphoma, and 24 known cancer types (Figure 3). The ribosomal protein genes that were up-regulated in CUP were also up-regulated in LAC.

**Figure 4:**
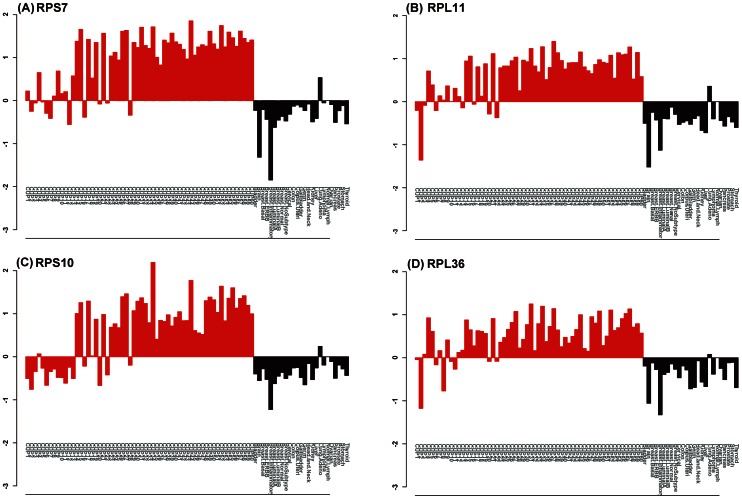
Relative expression levels for 4 ribosomal proteins. The relative expression levels of (A)RPS7, (B)RPL11, (C)RPS10, and (D)RPL36 were compared using individual CUP samples (n = 60), the mean expression levels of known cancer types, and a normal lymph node samples (n = 25). The asinh-transformed values for each gene were used for the calculations.

The relative mRNA expression levels of 4 ribosomal protein genes that were up-regulated in CUP (*RPS7*, *RPL11*, *RPS10*, and *RPL36*) were compared with the levels in normal lymphoma and 24 known cancer types (Figure 4). The 42 CUP samples that consistently contained large amounts of these mRNAs belonged to the larger CUP cluster, while the remaining 15 sample that showed relatively smaller amounts of these mRNAs belonged to the smaller cluster, as shown in Figure 2. As expected, the increased expressions of these mRNAs were also observed in LAC, but not in the other cancer types (Figure 4).

## Discussion

Accumulating data sets from gene-expression microarray analyzed for various types of tumors have enabled the establishment of organ- and tumor-specific expression profiles that improve precise prediction of primary site of CUP [9], [10], [14], [15]. Our official phase 2 study to corroborate the feasibility of CUP prediction using our algorithm is currently ongoing and will provide genes that exhibit unique expression pattern in CUP. A compelling theory to explain CUP is that the primary cancer is microscopic and may disappear because of marked apoptosis after seeding metastases that are able to proliferate into more significant tumors in different tissues [27]. As a high metastasis potential and vulnerability to apoptosis would explain the properties of CUP well, we first searched for genes related to metastasis and apoptosis among all the genes that were up-regulated by more than 2.5-fold in the CUP samples (M_CUP_(2.5) dataset).

Of the 14 up-regulated genes that were found (*S100A4, PRG1, S100A6, GSTP1, EIF5A, LGALS1, S100A11, PRKDC, VIM, CST3, TIMP1*, *YWHAZ*, *NEDD8*, *STK17A*), three (*S100A4*, *S100A6*, *S100A11*) belong to a group of S100 proteins involved in the Ca ^2+^ signaling network and regulate a variety of intracellular activities including cell growth and motility [28]. The expressions of these genes are observed in several epithelial tumors and have been linked to metastasis [29], [30]. *S100A4*, together with *VIM*, has also been used as an EMT marker [31]. The overexpression of *EIF5A* induces the EMT, thereby promoting the tumor metastasis of colorectal and hepatocellular carcinoma [32]. Serglycin, a gene product of *PRG1*, is a proteoglycan that has been functionally identified as a significant regulator of metastasis in nasopharyngeal carcinoma (NPC) [33]. The elevated expression of Serglycin in NPC cells can mediate the level of vimentin (*VIM*) expression, which is not only a marker of the EMT, but also has an important role in the regulation of cellular migration [31], [34]. Lewis lung carcinoma cells in mice show metastasis to the lung when the cells express Galectin-1 (Gal-1), a large carbohydrate-binding protein encoded by *LGALS1*, suggesting novel targeting strategies for Gal-1 in cancer [35].

Both metastatic cells and drug-resistant cells have similar gene expression patterns of survival-related molecules, suggesting that metastatic cancer may be difficult to treat because of resistance to anticancer drugs. DNA-dependent protein kinase (DNA-PK), a gene product of *PRKDC*, is one of the proteins up-regulated in several metastatic and drug-resistant cancer cells [36]. Because the up-regulation of DNA-PK was observed in the CUP patients in our cohort, who had never been treated with chemotherapy, DNA-PK may indicate essential resistance, rather than acquired resistance, to chemotherapy. GSTP1 has also been postulated in several cancer types to enhance the metastatic potential and the development of resistance to drugs that induce reactive oxygen species (ROS), such as paclitaxel and cisplatin [37], [38]. Other genes up-regulated in CUP also reveal a significant role in chemoresistance and may be linked to the metastatic potential. Breast cancer cells overexpressing TIMP-1, a well-known inhibitor of matrix metalloproteinase, exhibit a reduced sensitivity to the chemotherapeutic drugs paclitaxel and epirubicin through the activation of transcription factor NF-κB [39]. The knocked-down expression of 14-3-3 ζ, a gene product of *YWHAZ*, sensitizes head and neck cancer cells to chemotherapy [40]. A small molecule inhibitor of NEDD8 activating enzyme (NAE) may be active against tumors that are resistant to other chemotherapeutic agents [41].

Unlike the hitherto described genes, cystatin C (*CST-3*) and STK17A function as direct pro-apoptotic factors by antagonizing TGF-β signaling and by modulating ROS, respectively. Cystatin C has been shown to interact with the TGF-β type II receptor, thereby preventing TGF-β binding and subsequent EMT induction [42]. TGF-β has been accepted as a main initiator of EMT; however, NF-κB was recently found to promote EMT in some cells that are unresponsive to TGF-β because they lack functional SMAD4, representing an alternative pathway leading to EMT that can replace TGF-β signaling [43]. NF-κB signaling may predominately induce EMT in CUP. Both TIMP-1, which can activate NF-κB, and vimentin, which is activated by NF-κB, were among the genes (proteins) that were up-regulated in CUP as described above, making this hypothesis more likely [39], [43]. STK17A is up-regulated in response to oxidative stress in a p53-dependent manner [44]. Since STK17A is known as a positive regulator of the apoptotic pathway and its expression level in colorectal carcinomas is enhanced in lesions with lymph node metastasis, the apoptotic process could be involved in the node metastasis of carcinomas, including CUP [45].

Of the 15 down-regulated genes in the M_CUP_(2.5) dataset, *CD24*, *KRAS* and *DICER1* are of particular interest. CD24 is the most widely used marker, together with CD44, for identifying tumor-initiating cells in breast carcinomas. CD44^+^/CD24^−/low^ breast cancer cells have the ability to metastasize, since the enrichment of these stem-like cells is significantly observed in patients with positive lymph nodes [46]. A subset of kras mutant cancer cells exhibit “kras addiction” and have a differentiated epithelial phenotype. The induction of EMT has been shown to convert kras-dependent cancer cells to kras-independent cells, which do not require the continued expression of kras [47]. *Dicer1* functions as a haploinsufficient tumor suppressor gene [48]. Frequent loss of one allele of *Dicer1* has been observed in several different tumor types causing a global reduction of steady-state micro RNA levels that could be functionally suppressive to the oncogenesis and metastasis of CUP.

The increased expression of several ribosomal proteins was found in CUP. Whether these changes in expression are causally related to the generation of CUP is unknown. In some cases, the overexpression of ribosomal proteins, including RPL5, RPL11, RPL23 and RPS7 has been shown to suppress tumorigenesis [49], [50]. These proteins activate p53 by binding to MDM2 and inhibiting MDM2-mediated p53 ubiquitination and degradation in response to nucleolar stress (also called ribosomal stress). RPL11 and RPS7 were recently shown to be required for p53 activation induced by DNA-damaging agents [51], suggesting that these ribosomal proteins may play a crucial role in p53 activation in response to diverse stressors. Furthermore, neddylation, the process by which the ubiquitin-like protein NEDD8 is conjugated to its target, is essential for RPL11's role in the mediation of p53 signaling [49]. Interestingly, these two ribosomal proteins and NEDD8 were included in our M_CUP_(2.5) dataset. The tumor suppressor function performed by these proteins may be related to the vulnerability to apoptosis that CUP (at the primary site) exhibits as one of its properties.

For functional analyses of the identified genes, overexpression or knockdown experiments using appropriate cell lines would be plausible to pursue if the gene of interest confers change in growth or in metastatic ability to the cells. The metastatic process can be evaluated *in vitro* by monitoring cell invasion through Matrigel and adhesion of cells to plates, etc. Synthetic inhibitors specific to Gal-1, DNA-PK and 14-3-3 ζ have been developed [52]–[54]. Thus, it will be intriguing to investigate the effect of these inhibitors on the cells overexpressing the respective gene *in vitro* or *in vivo*, which may lead to targeted therapy for CUP.

To our surprise, the gene expression profile (GEF) of CUP closely resembled that of lung adenocarcinoma (LAC), which may simply reflect the relatively high metastatic potential of LAC. In a study using ^18^F-fluoro-2-deoxyglucose positron emission tomography (FDG-PET), the most commonly detected location of the primary tumor in patients with CUP was the lung [55]. In CUP, the primary cancer and its metastasis (-ses) behave very differently in respect to proliferation, leading to the assumption that the molecular profiles of CUP specimens from the two sites would differ. We are unable to compare these differences because the primary cancer is unidentifiable. A differential gene expression analysis using primary and metastatic tumor tissues from advanced lung cancer patients may provide some clues to this question.

In conclusion, we identified several genes that were up-regulated in CUP and that may contribute to the acquisition of a metastatic phenotype as well as resistance to anticancer drugs in many cases. Proapoptotic factors were also identified. The combinational effects of the multiple functions of genes that are highly expressed in CUP could be involved in regulating CUP behaviors, such as apoptosis and metastasis. Immunohistochemical-based or PCR-based validation of the candidate genes is needed to refine the molecular classification of CUP.

## Supporting Information

Figure S1 **Heatmap constructed as in** **Figure 1** **but excluding the** ***VAPA*** **gene.**
(TIF)

Figure S2 **Cluster dendrogram for each cancer type.** Clustering analysis was done using the Ward method and 77 ribosomal protein genes.
(TIF)
