# Supplementary figures and images for: A Microarray-Based Gene Expression Analysis to Identify Diagnostic Biomarkers for Unknown Primary Cancer
Source: PLoS One. 2013 May 9;8(5):e63249. doi: 10.1371/journal.pone.0063249 (PMC3650062; doi:10.1371/journal.pone.0063249)

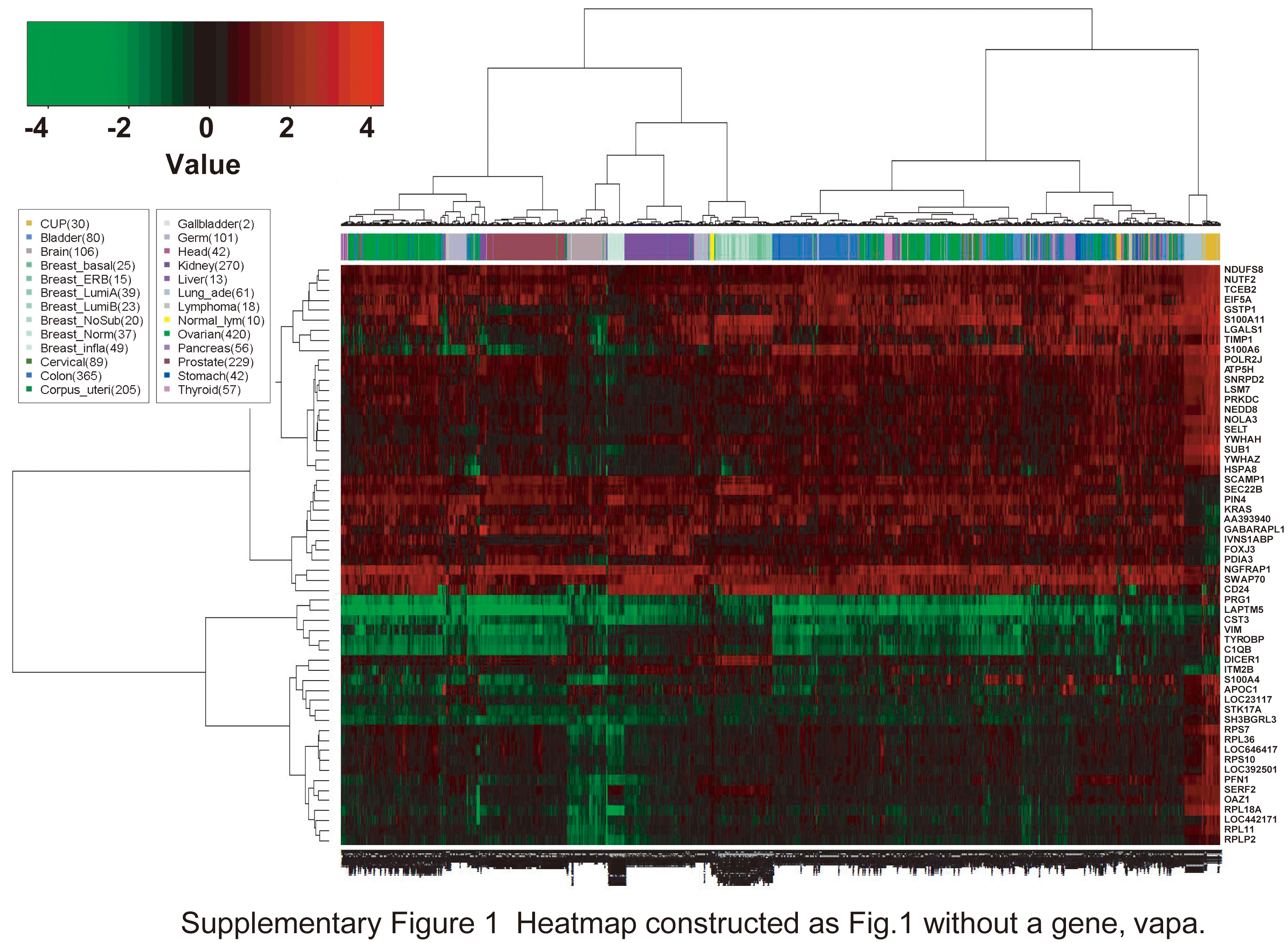

Supplement: Figure S1 — Heatmap constructed as in Figure 1 but excluding the VAPA gene. (TIF) [file pone.0063249.s001.tif]

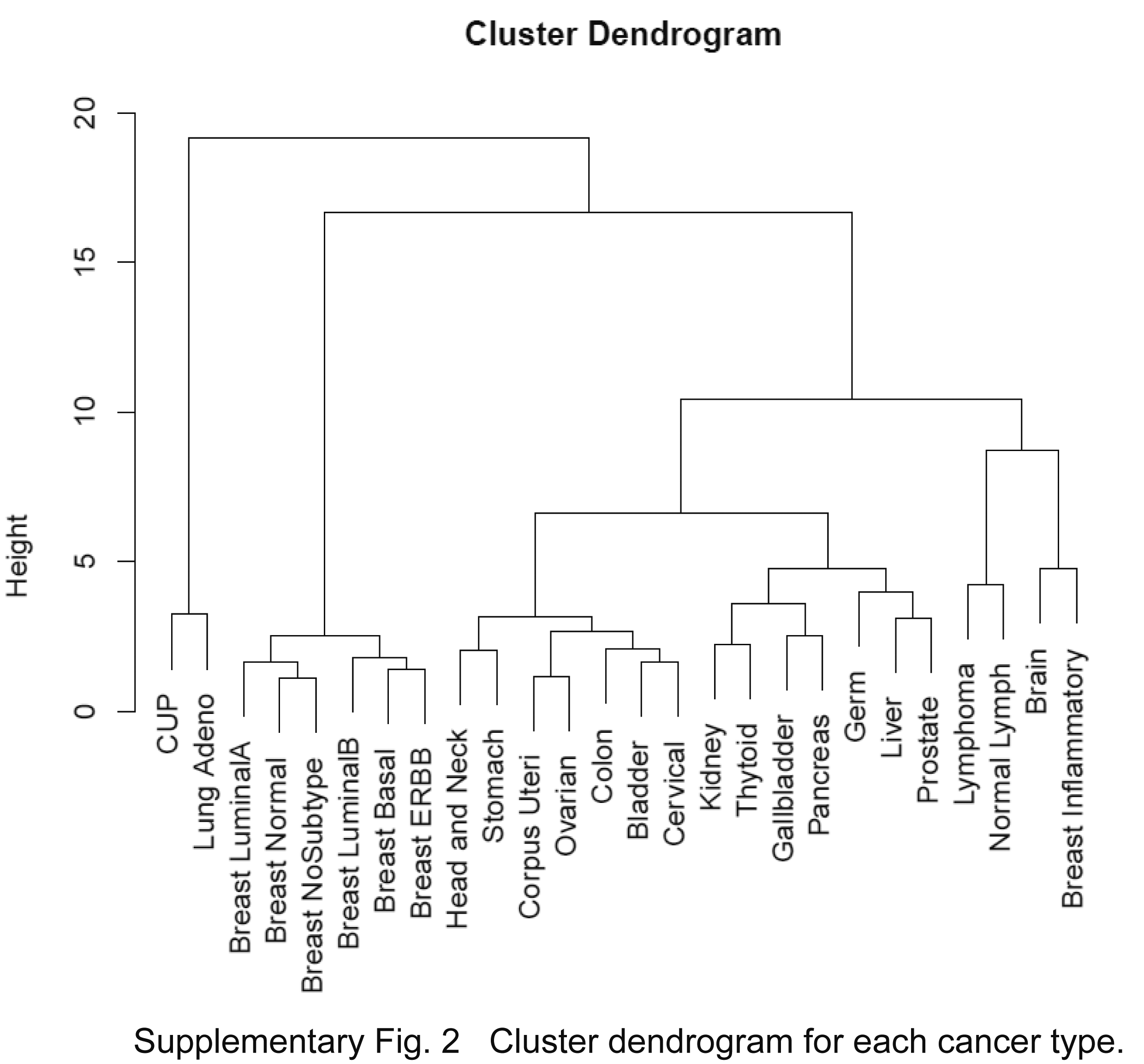

Supplement: Figure S2 — Cluster dendrogram for each cancer type. Clustering analysis was done using the Ward method and 77 ribosomal protein genes. (TIF) [file pone.0063249.s002.tif]
